# Dyspepsia

**Published:** 1893-12

**Authors:** Hugh Slevin


### DYSPEPSIA.

Dyspepsia means a difficulty in preparing the food eaten so that the nutriment can be extracted from it to supply the wants of the system.

If food is eaten rapidly, it is swallowed in large pieces and these are dissolved from without inwards by the juices of the stomach, as a lump of ice in a glass of water is dissolved from without inwards.

But this is a slow process, and if protracted beyond four or five hours, the food begins to ferment, to sour, causing belching, weight or heaviness at the pit of the stomach, sourness and a variety of other symptoms with which dyspeptics are very familiar.

One of the causes then of dyspepsia is eating too fast. If a person eats too much, the food remains in the stomach undigested, unmelted, undissolved, because there is not enough gastric juice to reduce it to the proper condition for yielding its nutriment; as so much ice may be put in a glass of water that after awhile it ceases to melt, and the food thus remaining unchanged for an unnatural time, it begins to sour as before, because the person has been eating too much. A man may have dyspepsia for the want of a sufficient amount of gastric juice to digest the food, although a very little food may have been eaten; hence the frequent complaint, "It makes no difference whether I eat much or little; the smallest quantity of anything distresses me." Such a person has dyspepsia in an aggravated form, from having had it for a long time. The limited supply of gastric juice is the result of poor or bad blood.

All the blood of a dyspeptic is bad because the food is imperfectly digested and the blood which it makes is imperfect, hence contains but a small amount of the elements which compose the gastric juice. The always successful remedy is to live out of doors night and day, exercising until a very little tired; then rest, exercise again until very hungry, until hungry enough to feel that plain bread and butter tastes deliciously; take a very small amount, such as by observation causes no discomfort whatever; then go on as before until very hungry again, take a little fresh meat at the next meal and a bit of bread crust; make the next or third meal of the day of berries, grapes, fruit or melons.

Persevering in this way, almost any dyspeptic will find himself getting better and better every day, because every breath of out-door air taken relieves the blood of some of its impurities, and every step, every motion of the hand or arm carries off out of the system, through the pores of the skin or otherwise a greater or less number of impure blood atoms; the blood being thus relieved of more of its impurities, makes a better quality of gastric juice ; this in turn digests the food more thoroughly, imparting more strength, giving a more vigorous appetite and the man is getting well before he knows it. The gizzard or stomach of a chicken when opened is found to contain grains of corn or wheat and small pebbles. The action of the muscles of the gizzard is to keep the grains of corn and sand in a constant circular motion, causing attrition, the sand being harder than the grains ; hence the action is a kind of grinder; so with the human stomach. In dyspepsia the muscles of the stomach are too weak to perform this grinding process and the human mill works so slowly that the food begins to decay before it is properly manipulated. All dyspeptics are weak ; every muscle of the body is weak and those of the stomach have their corresponding share of debility ; but they will get stronger inevitably by making better blood, by giving a better digestion in the way above described. Biliousness also causes dyspeptic symptoms.

When a man is bilious, it means that he has an excess of bile or a deficiency of it, which mean the same thing essentially, although it is not known that such a sentiment has ever been expressed in writing or in print. When a man has yellow jaundice he is bilious in the proper sense of the term, meaning that the bile has not been withdrawn from the blood by the natural and healty action of the liver, as shown by the yellowness of the skin. The blood is then so impregnated with bile, which is of a yellow color, that it tinges the skin and whites of the eyes. In this case there is a torpid liver, a sleepy liver ; it does not act, does not work. But the liver may withdraw the bile from the blood and accumulate it in the gall bladder, where it may be detained, and as a result, the discharges are of a lightish color and are attributed to a deficiency of bile.

Hence excess of bile and deficiency both mean that there is too much bile in the body either in the blood or in the gall bladder.

But the exercise already referred to will purify the blood of any of its unnatural constituents, of every kind of impurity, while careful eating imparts strength to make a better blood. Thus it is seen that whatever may be its symptoms, that is, the feelings, the manifestations to which it may give rise, the thing to be done is to get rid of the bad blood and supply a better in its place. The way to do this is to engage in out-door activities and so select the food as to enable the stomach to act upon it in such a manner that it may yield its nutriment to the system naturally.

HUGH SLEVIN, M. D.

